# Formulating Parasiticidal Fungi in Dried Edible Gelatins to Reduce the Risk of Infection by *Trichuris* sp. among Continuous Grazing Bison

**DOI:** 10.3390/pathogens13010082

**Published:** 2024-01-17

**Authors:** Rami Salmo, Cándido Viña, Izaro Zubiria, José Ángel Hernández Malagón, Jaime M. Sanchís, Cristiana Cazapal, María Sol Arias, Rita Sánchez-Andrade, Adolfo Paz-Silva

**Affiliations:** 1 Control of Parasites Group (COPAR, GI-2120), Department of Animal Pathology, Faculty of Veterinary, University of Santiago de Compostela, 27002 Lugo, Spain; rami.salmo@rai.usc.es (R.S.); candido.vina@rai.usc.es (C.V.); izaro88@gmail.com (I.Z.); joseangel.malagon@usc.es (J.Á.H.M.); cristiana.cazapal@usc.es (C.C.); rita.sanchez-andrade@usc.es (R.S.-A.); 2 Parasitology and Parasitic Diseases, Faculty of Veterinary, University of “La República” (Regional Litoral Norte), Salto 50000, Uruguay; sanchisjaime@gmail.com

**Keywords:** helminth control, sustainability, *Mucor circinelloides*, *Duddingtonia flagrans*, dry gelatin

## Abstract

Control of infection by gastrointestinal nematodes remains a big problem in ruminants under continuous grazing. For the purpose of decreasing the risk of infection by *Trichuris* sp. in captive bison (*Bison bison*) always maintained in the same plot, dried gelatins having ≥10^6^ chlamydospores of both *Mucor circinelloides* and *Duddingtonia flagrans* were given to them for one week, and at the end, fecal samples (FF) collected each week for four weeks were analyzed immediately. Feces taken one week prior to gelatin administration served as controls (CF). Eggs of *Trichuris* sp. were sorted into non-viable and viable, then classified into viable undeveloped (VU), viable with cellular development (VCD), or viable infective (VI). Ovistatic and ovicidal effects were determined throughout the study. In FF, viability of *Trichuris* eggs decreased between 9% (first week) and 57% (fourth week), egg development was delayed during the first two weeks, and VI percentages were significantly lower than in CF (*p* = 0.001). It is concluded that the preparation of gelatins with chlamydospores of parasiticidal fungi and their subsequent dehydration offer an edible formulation that is ready to use, stress-free to supply, and easy to store, as well as being well-accepted by ruminants and highly efficient to reduce the risk of *Trichuris* sp. infection among animals under continuous grazing regimes.

## 1. Introduction

Grazing management systems have returned in recent decades based on the increase in the cost of raw materials for feedstuff manufacturing. In addition, animal welfare became an important concern for consumers, who appreciate when animals can pasture outside for as long as possible, regarding the weather conditions. Different grazing regimes are applied, mainly to ensure adequate nutrition for the animals. Rotational pasturing appears very appropriate because nutritive needs are reached by moving animals to new prairies often (every two–three days) [1]. In contrast, it is generally assumed that continuous pasturing provides lower nutritional power because forage species cannot grow again quickly, making supplementation mandatory [2].

An important problem associated with pasturing regimes consists of herbivores that can become infected by taking infective stages of several parasites together with the herbage [3]. Hence, considerable efforts are required to control these pathogens responsible for important economic losses, reduction of the health status of the animals, and even their death [4]. Despite grazing animals receiving very successful deworming, reinfection occurs soon because of infective stages that develop in areas with vegetation. This underlines the need for proper information on the time the free-living stages can survive on the ground, and the population dynamics [5]. Rotational pasturing has been advised to reduce the risk of infection by certain parasites, based on the fact that herbivores might take advantage of grasslands with very low levels of contamination [6]. On the contrary, continuously grazed paddocks are regularly associated with elevated risk of infection, thus worsening the possibilities to control several parasitic infections. Constant and long-term use of the same area during winter causes an accumulation of invasive material and increased risk of helminth invasions [7].

The gastrointestinal nematodes are a very important group of helminths affecting herbivores, especially under pasturing conditions, and control is frequently performed by the regular administration of chemical anthelmintic drugs [8,9]. Most attention has been focused on infection by strongyles, and scarcely on others belonging to the genus *Trichuris* [10]. These species have a direct life cycle, and the infected individuals pass unembryonated eggs in the feces, which develop once in the soil until the infective stage appears (the egg contains a first-stage larva inside), approximately after 2–4 weeks [11]. After being swallowed, these eggs hatch in the intestine, and the released larvae move to the caecum and proximal colon, where the worms bury their narrow anterior end into the mucosa. Although many cases are asymptomatic, infection by *Trichuris* spp. can be responsible for bloody colitis, diphteritic caecitis, and ulcerative and necrotic lesions on the mucosa [12]. In the presence of high burdens of worms, anemia, dehydration, and jaundice evolve and cause death if appropriate treatment (benzimidazoles or macrocyclic lactones) is not administered [4,13]. Because of this, new strategies targeting the eggs of *Trichuris* in the soil appear essential for contributing to the control of this nematode, as a complement to the administration of efficient anthelmintics [14].

For the purpose of acting against different stages of helminths in the soil, the saprophytic filamentous fungus *Duddingtonia flagrans* and *Mucor circinelloides* have been simultaneously cultured in a submerged medium [15]. This formulation provides a very useful tool to trap and destroy the larvae of gastrointestinal nematodes as trichostrongylids, cyathostomin, or ancylostomatids by means of *D. flagrans* [9,16]. Additionally, *M. circinelloides* is capable of developing a type three effect on eggs of different species, consisting of eggshell injury, penetration of the hyphae inside, and embryo destruction. Previous investigations reported successful results when a blend of the chlamydospores of *M. circinelloides* and *D. flagrans* was formulated as aqueous solutions, and mixed with cereal grains or nutritional pellets [1,17,18,19]. In the current study, a novel formulation comprising the manufacturing of edible gelatins with chlamydospores of both fungi was developed. Then, edible gelatins were desiccated, and finally tested on captive bison maintained in a zoological garden under continuous pasturing.

## 2. Materials and Methods

### 2.1. Edible Gelatins with Fungal Chlamydospores

**Figure 1:**
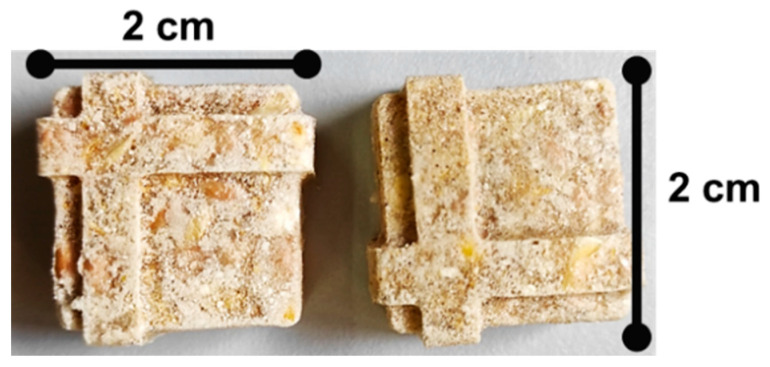
Gelatins were made from a blend of 10^7^ chlamydospores of both *M. circinelloides* and *D. flagrans,* and then dehydrated.

Two saprophytic filamentous fungi, *Mucor circinelloides* (CECT 20824; active against helminth eggs) and *Duddingtonia flagrans* (CECT 20823; helminth larvae antagonist)—isolated from soil and fecal samples of animal species and deposited in the Spanish Type Culture Collection (CECT, Valencia, Spain)—were co-cultured in a submerged medium until a quantity ≈ 10^7^ chlamydospores of every one was attained. This medium was added to edible gelatin powder, honey, and water (pending on registration). Once completely homogenized, the blend was heated under microwave for a brief period and placed into silicone molds (approximately 10 mL/each), quenched at 4–6 °C to enhance gelation, and then at −35 °C until frozen. Finally, the products were lyophilized (Figure 1), and packed into reusable plastic bags.

### 2.2. Effect of Drying on the Viability of Fungal Chlamydospores

Examination of the dried gelatins was conducted to ascertain if the procedure might decrease the number of chlamydospores. Every month for six months, 30 gelatins were freeze-dried, then rehydrated by submerging each one into 10 mL water at 37 °C, and stirred until completely melted. Finally, five 25 µL-aliquots of each solution were taken, and their spores were counted in a Neubauer chamber under an optical microscope at 20× and expressed as numbers per mL. The reduction of the number of chlamydospores during the drying process was estimated as follows:

Reduction drying (%) = [1 − (Counts _DRY GELATIN_/Counts _GELATIN_)] × 100.

The analysis of the dried gelatins revealed that a percentage of 5–7% chlamydospores were lost during the lyophilization, so this formulation enabled that every individual received ≥10^6^ chlamydospores of each parasiticide fungus/piece.

### 2.3. Captive Bison

**Figure 2:**
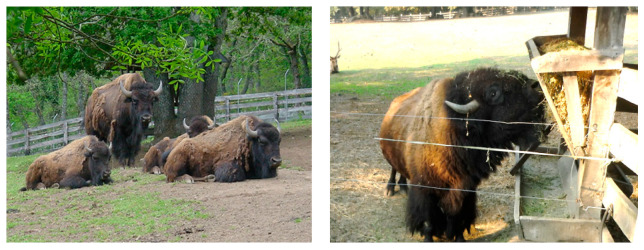
Captive bison under continuous grazing and passing eggs of *Trichuris* sp. in their feces received dried gelatins containing a blend of 10^6^ chlamydospores of both *M. circinelloides* and *D. flagrans* each (Marcelle Natureza Zoological Park, NW Spain).

Seven captive bison (*Bison bison*) maintained in the Marcelle Natureza Zoological Park (Outeiro de Rei, Lugo; 43°4′14.71″ N, 7°37′53.50″ W; Spain) were utilized in the current study (Figure 2). These ruminants are maintained under continuous grazing in a 1-Ha fenced paddock with trees and grass. Nutritional supplementation consisting of pelleted feed is provided every 2 days, as well as hay during the winter. Feces is removed manually every 2–3 days prior to the visitors entering the park. Deworming involves the quarterly administration of anthelmintics (albendazole or ivermectin).

### 2.4. Experimental Design

During one week, fecal samples were taken directly from the soil and considered as controls (CF, control feces). After being homogenized by mixing vigorously, a quantity of four grams was placed into 16 plastic boxes with holes in each wall to allow aeration, then a plastic cover was put on top and it was moved to a grassland for four weeks [20].

For a period of one week, every bison was given one gelatin daily, and at the end, feces were collected and labelled as FF (feces with fungi). Again, fecal samples were homogenized, and four grams were deposited into 24 plastic boxes, which proceeded as explained above. In order to guarantee that each bison received the corresponding dosage of chlamydospores, one dry gelatin was mixed with ca. 10 g nutritional pellets and put into the feeder.

### 2.5. Evaluation of the Strategy

The fecal contents of 4 CF boxes and 6 FF boxes were analyzed weekly by means of a coprological flotation test for the purpose of detecting the presence of parasites [20]. Briefly, feces were emulsified in 42 mL water, stirred and filtered through a 150 µm mesh, then collected into 12 mL tubes. After centrifuging at 2000 rpm for 5 min, supernatants were discarded, and the sediments were resuspended into a NaCl saturated solution (ρ = 1.25 g/mL) and observed under an optical microscope (10×–20×) by placing 20 µL between a glass slide and coverslip, until a minimum of 150 eggs of *Trichuris* sp. were examined. This analysis was conducted in replicate for each fecal sample.

**Figure 3:**
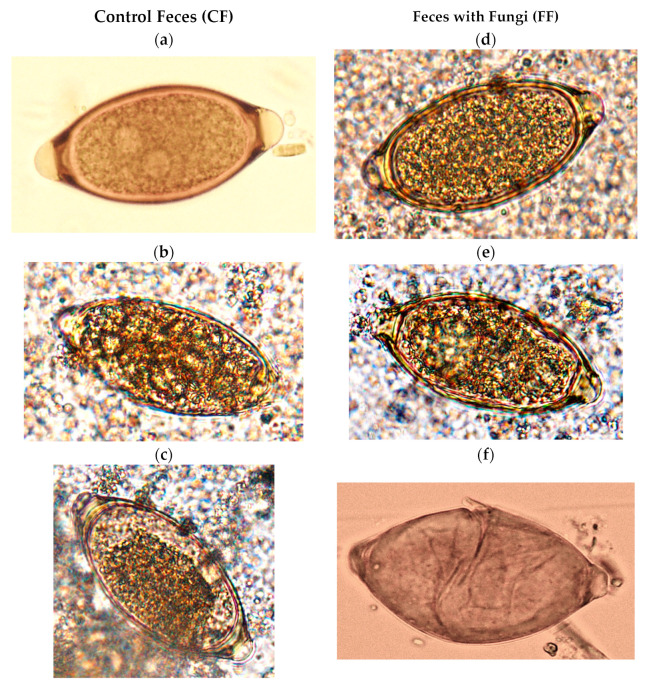
Evolution of eggs of *Trichuris* sp. in feces of bison kept under continuous grazing. CF (controls): samples collected prior to providing bison fungal chlamydospores. (**a**) Viable unembryonated, with two nuclei; (**b**) viable with cellular development (VCD); (**c**) viable with cylindrical larva (VI, infective). FF: samples taken after giving bison a blend of chlamydospores. (**d**) Viable undeveloped (VU); (**e**) cytoplasm vacuolization (non-viable); (**f**) empty, with eggshell disrupted (non-viable).

The effect of the parasiticide fungi on the eggs of *Trichuris* spp. was based on their ability to destroy them or damage them permanently (ovicide effect), as well as to influence the capability of the eggs to develop to the infective stage (ovistatic effect). In this way, eggs of *Trichuris* were sorted firstly into non-viable and viable, with respect to the observation of at least one disturbance, including shell disruption or contraction, cytoplasm vacuolization, or larval immobility when light stimulated (presence = non-viable; absence = viable) [21] (Figure 3). The ovicidal effect or viability reduction (VR) was assessed weekly by comparing the number of viable eggs in the feces with fungi (FF) and those in the controls (CF) (1):

(1) $$\mathrm{VR}\;(\%)=\left[1-\left(\frac{FF\;\mathrm{Viable}\;\mathrm{eggs}}{\mathrm{CF}\;\mathrm{viable}\;\mathrm{eggs}}\right)\right]\times \;100$$

Assessment of the ovistatic effect comprised the evaluation of the development of the eggs in the FF in comparison to the controls (CF). For this purpose, viable eggs were further classified as undeveloped (VU), with cellular development (VCD), or infective (VI) (Figure 3) [18]. The ratios for the different *Trichuris* sp. ova development stages in CF and FF were estimated as follows (2):

Ratio = % Eggs_CF_/% Eggs_FF_ (2)

Finally, the Soil Contamination Index (SCI) was calculated for FF and CF to get information about the risk of infection in the soil (3):

SCI (%) = (% Viable Eggs × % of Eggs with L1)/100(3)

### 2.6. Statistical Analysis

The analysis by means of the Kolmogorov–Smirnov probe showed that data regarding the numbers of viable eggs, VU, VCD, and VP were not normally distributed (*p* < 0.05), and the Levene probe confirmed that the variances were not homogeneous (*p* < 0.05). Accordingly, the nonparametric Mann–Whitney U test was performed (significance level *p* < 0.05). All of the probes were conducted by using the IBM^®^ SPSS^®^ Statistics for Windows, version 21 (IBM Corporation, Armonk, NY, USA).

## 3. Results

### 3.1. Variations on the Viability of Eggs of Trichuris sp.

**Figure 4:**
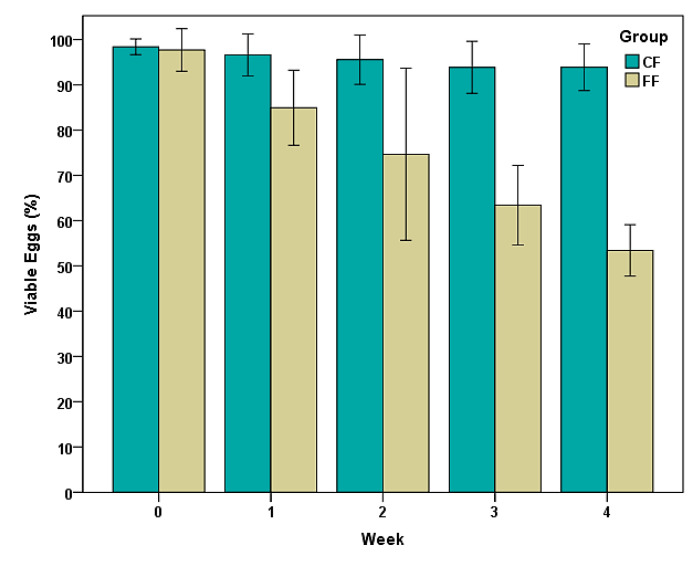
Percentages of viable eggs of *Trichuris* sp. in feces of bison under continuous pasturing. FF: samples taken after giving bison a blend of chlamydospores; CF (controls): samples collected prior to providing bison fungal chlamydospores. Points represent the percentage average ± 2 SD.

As illustrated in Figure 4, minor variations were observed in the percentages of viable eggs of *Trichuris* sp. in CF (control fecal samples), and values higher than 95% were achieved throughout a period of four weeks. In FF (fecal samples taken one week after bison received daily gelatins with fungal chlamydospores), viability decreased from 99% to 53% during the same interval, which means a viability reduction of 46%. These differences were statistically significant (Z = −5.734, *p* = 0.001).

### 3.2. Evolution of Viable Eggs of Trichuris sp.

**Figure 5:**
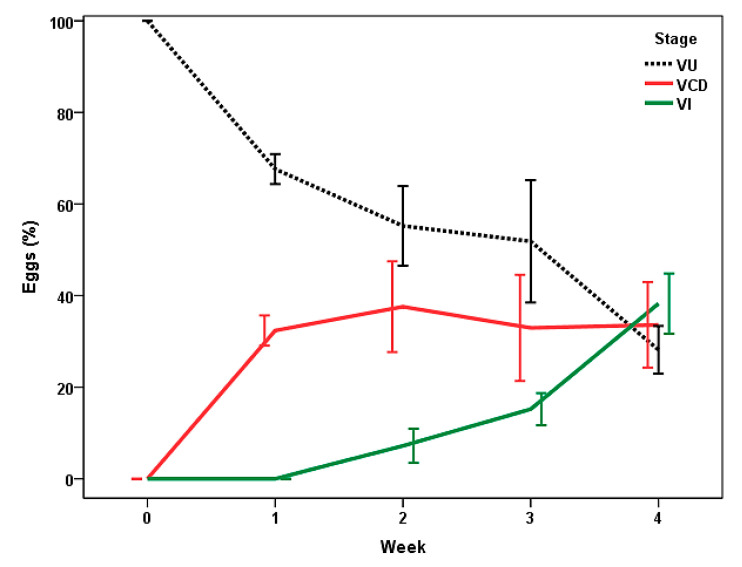
Stages of viable eggs of *Trichuris* sp. in feces of bison under rotational grazing (CF). VU: unembryonated (zygote); VCD: with cellular development; VI: infective. Points represent the percentage average ± 2 SD.

By the first week of the study (Figure 5), cellular development was recorded inside the eggs of *Trichuris* sp. in CF, with values around 32%. Infective eggs (VI, containing a L1 inside) were identified from the second week, with the maximal counts at the end of the study (near to 40%).

**Figure 6:**
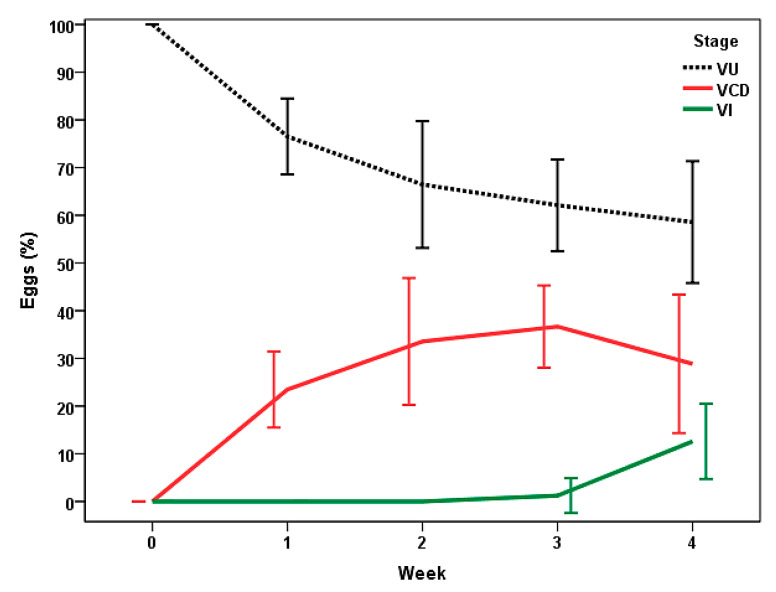
Stages of viable eggs of *Trichuris* sp. in feces of bison kept under continuous pasturing, after receiving dry gelatins containing a blend of chlamydospores of *M. circinelloides* and *Duddingtonia flagrans* (FF). VU: unembryonated (zygote); VCD: with cellular development; VI: infective. Points represent the percentage average ± 2 SD.

In the FF (Figure 6), about 25% of the eggs showed cellular development at the first week, increasing to the third week (≈37%). The presence of infective eggs (VI) was detected from the third week (1%), and numbers near 13% were attained at the end of the study.

Statistical differences were demonstrated between the two groups for VU (Z = −3.062, *p* = 0.002) and VI (Z = −3.806, *p* = 0.001).

No problems related to gelatin preparation, dehydration, or storage were recorded throughout the study. Dry gelatins containing the fungi were well-accepted by all the bison, and no trouble regarding their intake was observed.

### 3.3. Effect of Ingestion of Fungal Chlamydospores on Eggs of Trichuris sp.

Regarding the analysis of the ovistatic effect, the ratios for the eggs of *Trichuris* between CF and FF are summarized in Table 1. At the end of the study, the number of VU in FF duplicated in relation to CF, whereas the counts of VI decreased by one-third.

According to the analysis of the ovicidal effect, counts of viable eggs of *Trichuris* in CF were 1.8 times higher than in FF by the end of the study (Table 1). A significant 44% reduction of egg viability in FF, with respect to CF, was demonstrated after four weeks (Z = −3.710, *p* = 0.001) (Table 2).

Table 3 shows that the risk of soil contamination by *Trichuris* sp. was five times higher in CF (feces taken before giving chlamydospores to the captive bison) than in FF (samples collected after bison ingested the fungal chlamydospores).

## 4. Discussion

Anthelmintic deworming provides successful results against gastrointestinal nematodes, but animals become infected again by *Trichuris* and others, especially when reared under pasturing regimes, because of the existence of infective stages in the soil [22].

Without depending on their stage (zygote, morula, blastula, larva), eggs of *Trichuris* spp. can survive for years in moist and shady areas [3,4]; thus, it appears quite plausible that minimizing pasture contamination by eggs might represent a major and helpful component of the control of infection [23]. In the current study, a blend of chlamydospores of two fungi with parasiticide activity, *M. circinelloides* and *D. flagrans,* was given to bison kept under continuous grazing and passing eggs of *Trichuris* spp. in their feces. Two weeks later, egg-viability decreased 1.3-fold, and 1.8-fold at the fourth week. These results point to the ovicide effect that is achieved in the presence of parasiticide fungi in the feces of captive bison, which is in agreement with previous studies conducted against eggs of *Trichuris* sp. in feces of dromedaries captive in a zoo [20].

Once in the soil, eggs of *Trichuris* undergo different stages, and after 2–4 weeks, depending on environmental conditions (temperature and humidity), a first instar larva is formed inside. Infection occurs only when the infective stages are swallowed, which means that ingestion of the other egg stages poses no risk to the animals. For this reason, the period of time until the appearance of L1 is of great importance. In the present study, the presence of L1 in the control feces (without chlamydospores) was recorded since the second week, in congruence with previous research [14]; in addition to this, more than two-fifths of the eggs exhibited development inside at that moment. In contrast, only 1% of the eggs in the feces with fungi became infective by the third week, and two-thirds still remained undeveloped as a zygote at that time, which represents double of those in the absence of fungi (controls). These results highlight that an elevated ovistatic effect can be attained in feces by giving bison a blend of chlamydospores of *M. circinelloides* and *D. flagrans*.

Most of the information about the usefulness of fungi to control parasitic infections has been acquired by providing the animals’ chlamydospores of *D. flagrans* [24,25]. Recently, it has been demonstrated in vitro that there is compatibility and nematicidal activity of *Pochonia chlamydosporia* (ovicide) and *Arthrobotrys cladodes* (larvicide), or *P. chlamydosporia* and *Monacrosporium sinense* (larvicide), against larvae of gastrointestinal nematodes affecting bovids [26,27,28], as it was found formerly with *M. circinelloides* and *D. flagrans* [15]. In the present research, this blend of chlamydospores confirmed that the parasiticidal spectrum notably increases because of the antagonistic effect exerted simultaneously on eggs and larvae developing in the feces and in the soil [28].

One interesting question associated with the use of parasiticidal fungi on animals relies on their administration. By means of aqueous solutions [18,29,30], handmade [31] or industrially manufactured nutritional pellets [19], or cereal flour [32,33], spores and/or mycelium of several fungi have been given to different animal species. These formulations should not only be effective, but easy to prepare, preserve, and apply. To comply with these objectives, in the current study chlamydospores of *M. circinelloides* and *D. flagrans* were formulated as edible gelatins, then dried to enhance their preservation. In this way, a final product was obtained after a simple procedure that can be kept for long periods without refrigeration or freezing, and is very handy to give to the animals, alone or mixed with the feed.

Apart from management of pastures, there is scarce information about preventing infection by gastrointestinal nematodes (including trichurosis), therefore anthelmintic treatment is highly utilized. This measure does not affect the survival and/or development of the parasites in the soil until they reach the infective stage, so the chance of infection remains high or even increases. It must be emphasized that in the present research, egg viability dropped to less than half, and risk of soil contamination by *Trichuris* sp. reduced five times in feces containing parasiticide fungi. Rotational grazing is commonly advised to reduce the risk of infection by certain helminths, based on low levels of contamination that are attained. This entails that some periods of grazing exclusion help to limit the repeated defoliation of the species, but a high number of prairies could be involved, which would make it difficult to implement [34]. As for confined animals in zoos, rotation of pastures is not possible if they are kept in fenced plots, due to the stress involved in moving them from one to another, the possibility of establishing visual contact with other animal species that are their natural predators, or of hearing them. Hence, taking into account the present study as well as previous research also referring to captive ruminants always fed in the same plot [7,17,18], data obtained after the administration of chlamydospores of *M. circinelloides* and *D. flagrans* to bison could be directly extrapolated to domestic ruminants kept in continuous grazing, and the risk of infection would be expected to decrease significantly. In the last decade, the ability of condensed tannins to reduce the levels of infection by gastrointestinal nematodes has encouraged a promising investigation comprising ruminants feeding on tannin-rich plants [35,36].

Finally, it is important to note that, by the action of rain or earthworms, the eggs of *Trichuris* (and other gastrointestinal nematodes) can be leached and, therefore, could be found from the soil surface to depths of up to 10 cm [37]. Consequently, the oral administration of spores of parasiticide fungi offers a very helpful procedure to ensure that the antagonistic agents are in close contact with the eggs, regardless of their position. This has a significant advantage over other application methods, such as spraying directly on soil or feces [20,21,30].

## 5. Conclusions

The preparation of edible gelatins with chlamydospores of *M. circinelloides* and *D. flagrans* and their subsequent dehydration offers an edible formulation that is ready to use, stress-free to supply, and easy to store. Based on the fact that this formulation is well-accepted by ruminants and highly efficient to minimize the risk of *Trichuris* sp. infection, it is strongly recommended among animals under continuous grazing regimes.

## Figures and Tables

**Table 1:** Ratios in the eggs of *Trichuris* sp. between CF and FF.

Week	0	1	2	3	4
**VU**	1	0.9	0.8	0.8	0.5
**VCD**	-	1.4	1.1	0.9	1.2
**VI**	-	-	-	15	3
**Viable Eggs**	1	1.1	1.3	1.5	1.8

VU: unembryonated (zygote); VCD: with cellular development; VI: infective. FF: samples taken after giving a blend of chlamydospores to bison kept under continuous grazing; CF (controls): samples collected prior to providing bison fungal chlamydospores.

**Table 2:** Values of reduction of viability (VR) of eggs of *Trichuris* sp. in feces of bison under continuous grazing provided a blend of chlamydospores of *M. circinelloides* and *D. flagrans* (FF).

Week	1	2	3	4
**VR (%)**	12	22	33	44

FF: samples taken after giving a blend of chlamydospores to bison kept under continuous grazing.

**Table 3:** Values of Soil Contamination Index (SCI) by *Trichuris* sp.

Week		0	1	2	3	4
**SCI (%)**	CF	0	0	7	14	36
	FF	0	0	0	1	7

FF: samples taken after giving a blend of chlamydospores to bison kept under continuous grazing; CF (controls): samples collected prior to providing bison fungal chlamydospores.

## Data Availability

The data presented in this study are available on request from the corresponding author.
